# Extrahepatic Textiloma Long Misdiagnosed as Calcified Echinococcal Cyst

**DOI:** 10.1155/2013/261685

**Published:** 2013-02-26

**Authors:** Federico Cattaneo, Massimo Graffeo, Enrico Brunetti

**Affiliations:** ^1^Division of Infectious and Tropical Diseases, University of Pavia, IRCCS S. Matteo Hospital Foundation, WHO Collaborating Center for Clinical Management of Cystic Echinococcosis, 27100 Pavia, Italy; ^2^Unit of Gastroenterology and Digestive Endoscopy, Poliambulanza Hospital Foundation, 25124 Brescia, Italy

## Abstract

Textiloma or gossypiboma is a retained surgical swab in the body after an operation and is a complication that can remain undetected for many years and may represent a diagnostic dilemma depending on its location. It may be confused with several focal lesions and an accurate history taking, combined with clinical and instrumental data, is key to suspecting the diagnosis. We report a case of abdominal textiloma that was initially misdiagnosed as echinococcal cyst and discuss the differential diagnosis based on sonographic features and the WHO-IWGE classification.

## 1. Introduction

The term textiloma or gossypiboma indicates a gauze pad that is left behind in a body cavity during a surgical operation. 

This type of complication is uncommon but may cause significant morbidity (close to 50%) and a high mortality rate (11–35%) [1–3]; furthermore, it may represent a diagnostic dilemma with important legal implications [3].

The incidence of textiloma is between 1 in 100 and 1 in 3000 for all surgical procedures [4–7] and 1 case in every 1000–1500 abdominal operations (most commonly complicated by its occurrence) per year [4, 6, 8]. The real incidence, however, may be higher because case numbers are calculated only based on malpractice claims and because of fear of legal repercussions [9].

Therapy consists of the removal of the textiloma on laparoscopy or laparotomy with treatment of complications. Only reoperation allows a definitive diagnosis [1, 10, 11].

We report a case of textiloma initially misdiagnosed as echinococcal cyst and discuss differential diagnosis based on sonographic features of the lesion. 

## 2. Case Report

A 55-year-old Italian woman was referred to our clinic for a suspected echinococcal cyst of the liver. 

She had type 2 diabetes mellitus, *β* thalassemia trait, dyslipidemia, and cervical arthrosis and had undergone cholecystectomy in 1973. 

In April 2006 an abdominal ultrasound performed at another hospital showed an enlarged liver with regular edges, steatosis, and a focal lesion 9.5 × 7.5 cm in diameter described as a cyst (suspected parasitic) partially solid within segments VI and VII. A hyperechoic area consistent with calcification was also found. The patient reported that she was aware of the cyst but could not provide any documentation. She was asymptomatic and stated that she had always refused to undergo further clinical investigations.

The patient had three further hospitalizations in the same hospital for poorly controlled diabetes in October 2007, May 2009, and March 2010 during which repeated ultrasound scans showed no changes in the lesion, diagnosed as a morphologically stable parasitic cyst.

During the third hospital stay she underwent a CT that showed a “mass with expansive growth with regular edges 11 cm in diameter, with liquid and calcified content external to the liver parenchyma.” Serology for cystic echinococcosis (complement fixation test) tested negative.

**Figure 1:**
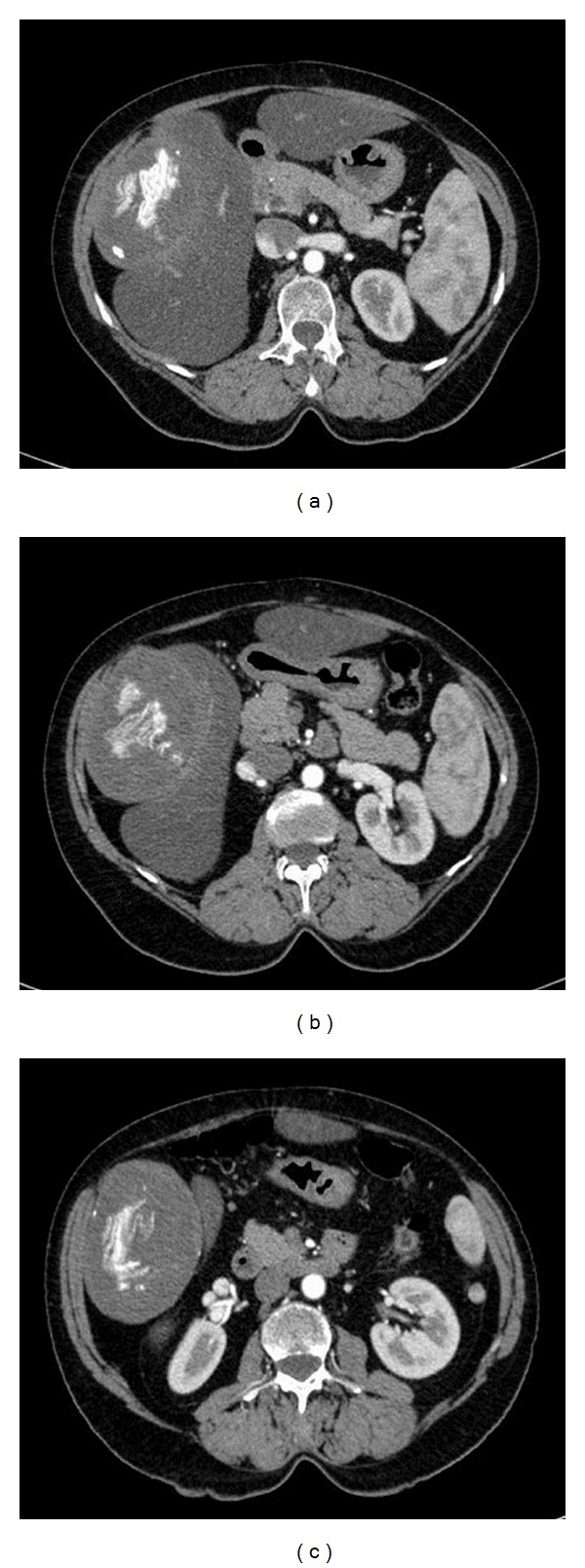
Gossypiboma seen at TC scan. There is an extra hepatic cystic mass with central calcifications.

On April 28, 2010, the patient was evaluated again in a hepatology clinic in a different town, with a new serology returning borderline result (1 : 80 with indirect hemagglutination - IHA). The CT scan done on 03/12/2010 during the third hospitalization was reviewed and considered not suggestive of a parasitic cyst (Figure 1) so the patient was referred to the Division of Infectious Diseases of the Policlinico San Matteo in Pavia for a second opinion. 

**Figure 2:**
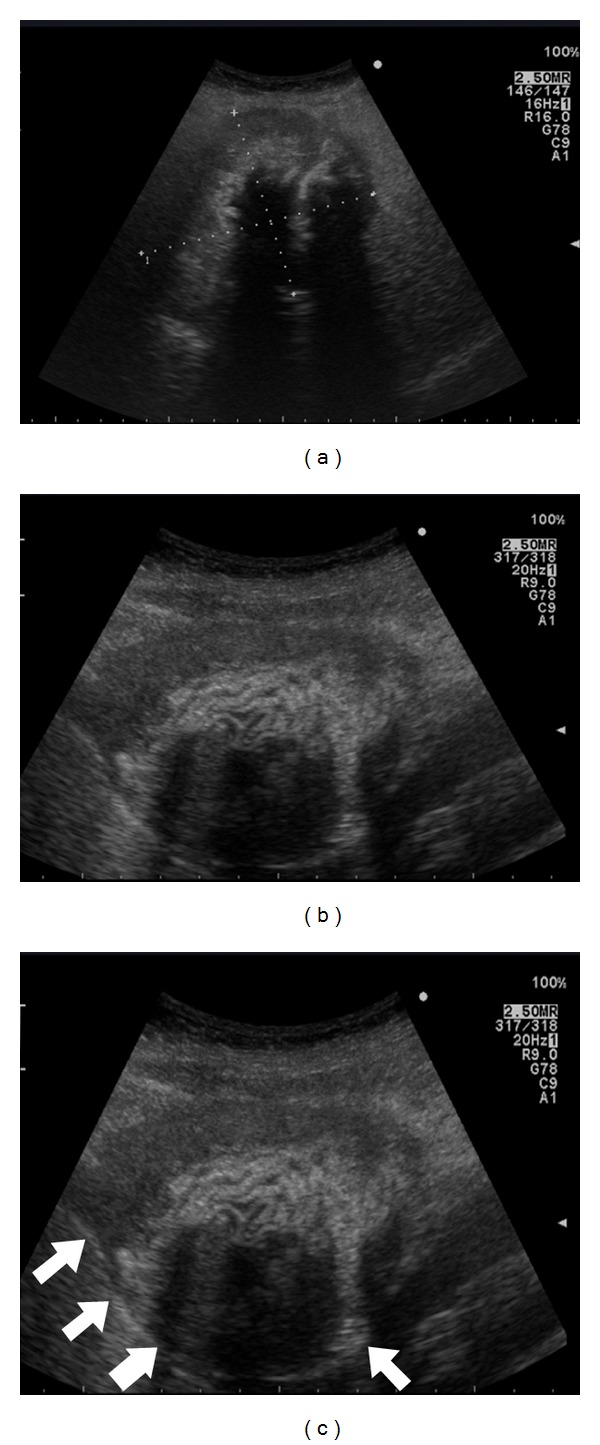
Extrahepatic gossypiboma seen at US scan. The arrows show the hepatic margin (c).

**Figure 3:**
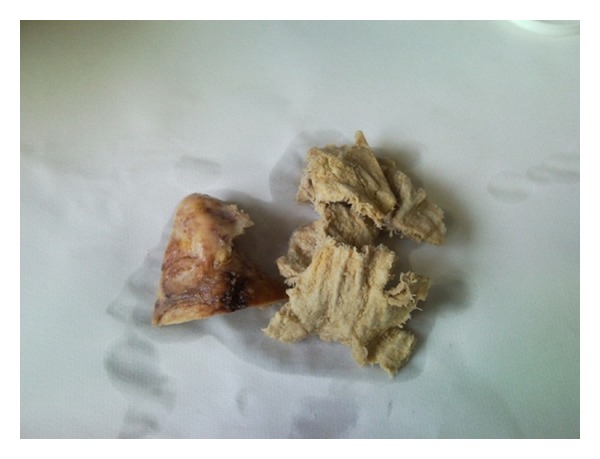
Pathologic findings. Gauze and liver fragment.

On May 5, 2010 the patient had a new serology for CE tested in Pavia with IHA (Cellognost*-Echinococcosis; Siemens Healthcare-diagnostics; Marburg, Germany. Cutoff for positivity >1 : 64) and ELISA (Echinococcus IgG K7621; RIDASCREEN; Darmstadt, Germany. Cutoff for positivity 0,5 until 2005 then 1,1) and returned negative. A new ultrasound scan was performed by a clinician with a long-standing experience in CE (EB) and for the first time the suspicion of a foreign body (gauze pads left in the patient's abdomen from cholecystectomy in 1973) was raised. Because the lesion had features that pointed to a textiloma (Figure 2), the patient was operated on and the clinical suspicion was confirmed (Figure 3). 

## 3. Discussion

A textiloma can cause two types of reactions: a fibroblastic reaction, as with a foreign body reaction, when an aseptic process begins (asymptomatic/palpable mass), or an exudative reaction which often leads to abscess (pain, fever, weight loss, fistula, intestinal obstruction or perforation, ileus caused by surgical adhesions, granulomatous peritonitis, and sepsis) [1, 13]. Therefore, clinical presentation of gossypiboma is variable and depends on the location of the retained swab and on the type of biological reaction.

Textiloma can be discovered in the first days after surgery or can remain asymptomatic (hence undiagnosed) for many years and be discovered accidentally. 

In our case the gauze pads left in the patient's body produced no symptoms for an exceptionally long time (37 years), whereas in the available literature gauze pads have remained undetected on average for 6 to 9 years [14–16]. The longest reported interval between the probable causative operation and the diagnosis of retained surgical spoon is 43 years [3, 4, 14, 17].

A thorough medical history, which can reveal previous surgery, and lab tests together with imaging (ultrasonography, computed tomography, or magnetic resonance) are crucial elements for diagnosis of textiloma [11].

**Figure 4:**
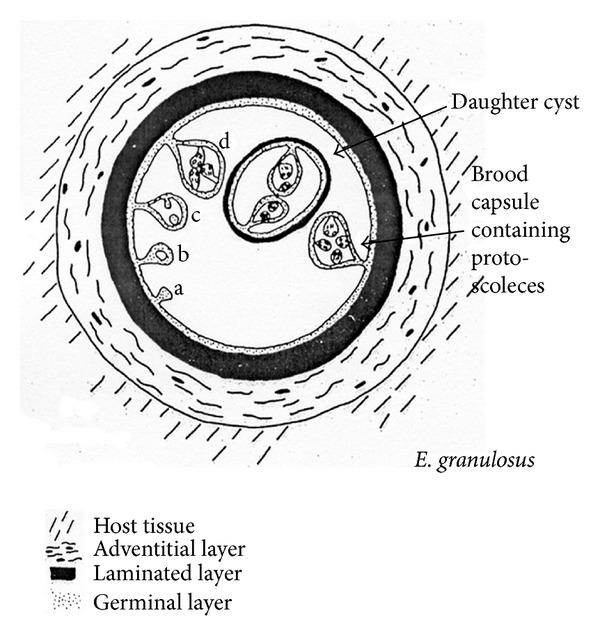
Cyst's structure from WHO/OIE Manual on Echinococcosis in Humans and Animals: A Public Health Problem of Global Concern, edited by J. Eckert, M. A. Gemmell, F. X. Meslin, and Z. S. Pawłowski.

Differential diagnosis includes tumor; cysts, parasitic and otherwise; hematoma, and inflammatory tumor [4, 13, 18, 19], we ruled out CE based on the knowledge of the echinococcal cyst structure (Figure 4). The origin of the patient from an endemic area (Southern Italy) and the negative serologic tests, that would have been in accordance with the cyst being inactive (although calcifications can be found at virtually any stage of the cyst history [20, 21]), were two confounding factors.

In our case, the correct diagnosis was made in two steps: 

The “calcifications”, however, were not seen at the periphery of the “cyst” as seen in CE, but at the center (Figures 1 and 2). 

**Figure 5:**
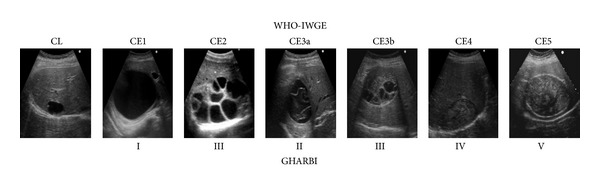
WHO-IWGE international standardized CE classification modified by Brunetti et al. International CE Workshop in Lima, Peru, 2009 [12]. Cystic echinococcosis: chronic, complex, and still neglected. PLoS Negl Trop Dis. 2011; 5(7):e1146. Note the external calcification of CE5.

As seen in the WHO-IWGE ultrasound classification images (IWGE-WHO 2003) [22] (Figure 5) cysts are either fluid filled (CE1, CE3a) or filled with daughter cysts (CE2) or matrix with (CE3b) or without (CE4, CE5) daughter cysts and calcifications are seen around the cyst but not inside [20]. 

**Figure 6:**
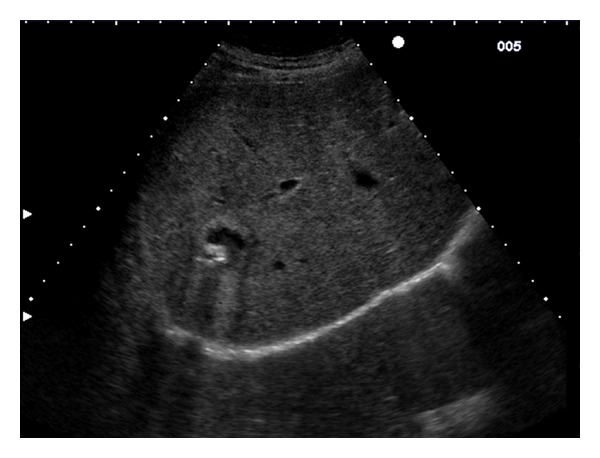
Ultrasound appearance of a brucellar abscess. A central dense calcium deposit is also observed with acoustic shadowing.

To our knowledge, the only focal infectious lesion that has a central calcification is brucellar abscess (Figure 6) [23, 24].

On closer inspection, though, parallel, wavy hyperechoic lines strongly reminiscent of a gauze pad were seen (Figure 2), which were in line with the patient's previous surgical intervention on the liver and helped exclude CE. 

These two elements were enough to make a diagnosis with CT scan not adding much besides confirming the extrahepatic location of the lesion. In addition, US performs better than CT in staging the cyst [21] and stages as defined by US have been shown to match cyst activity [25].

**Figure 7:**
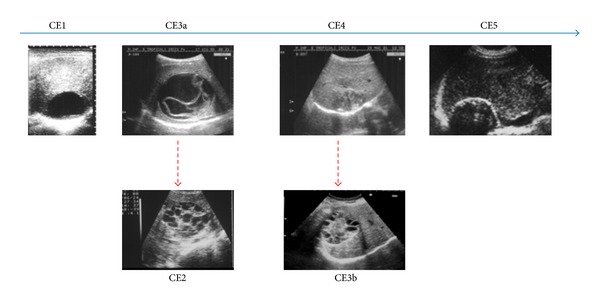
Cyst stages are different phases of the “natural history” of an echinococcal cyst. The upper row depicts the progressive involution (L to R) of the cyst whose cavity is gradually filled by pseudocaseous inflammatory material. Dotted lines indicate relapses from previously transitional and inactive stages, with the growth of new daughter cysts.

Differential diagnosis should take into account the different CE stages, understood not simply as different types of cysts, but as different phases in the natural history of a chronic disease (Figure 7) [12].

Although there are reports of gossypibomas taken for CE because of serpiginous lines mimicking the waterlily sign of CE3a produced by the detached endocyst [11], our case has none of this and was in the end rather easily diagnosed based on US findings alone [21].

This case underlines that knowledge of the main sonographic features of echinococcal cyst should be part of the differential diagnosis and, in case of suspected echinococcal cyst, contacting a specialist from a referral center may shorten the time to diagnosis.
